# The Mixed Impact of Environmental Regulations and External Financing Constraints on Green Technological Innovation of Enterprise

**DOI:** 10.3390/ijerph191911972

**Published:** 2022-09-22

**Authors:** Mingyue Wang, Junbi Zhou, Xiaojin Xia, Zitong Wang

**Affiliations:** 1 Institutes of Science and Development, Chinese Academy of Sciences, No. 15, Zhongguancun Beiyitiao, Haidian District, Beijing 100190, China; 2 National Science Library, Chinese Academy of Sciences, No. 33, North 4th Ring Road West, Zhongguancun, Beijing 100190, China; 3 Tianjin Academy of Science and Technology for Development, No. 138 Xinkai Road, Hedong District, Tianjin 300011, China; 4 School of Public Policy and Management, University of Chinese Academy of Sciences, No. 19, Yuquan Road (A), Shijingshan District, Beijing 100049, China

**Keywords:** environmental regulation, green technological innovation, external financing constraints, inverted *U*-shaped relation, manufacturing firms

## Abstract

Green technological innovation is an important force for high-quality economic development and high-level ecological environment protection. Environmental regulation and market financing are important factors affecting enterprise green technological innovation, while the relationship between environmental regulation and enterprise green technological innovation is most likely to be nonlinear. Additionally, this impact may be moderated by market financing. Based on the data of 2278 manufacturing enterprises in China, this article intends to empirically test the nonlinear relationship between environmental regulation and enterprise green technological innovation. Green technological innovation is divided into green process innovation and green product innovation. Based on this, the analysis of the heterogeneous impact of environmental regulations on different types of green technology innovation is implemented. Moreover, the moderating effect of external financing constraints on the relationship between environmental regulation and green technological innovation is further discussed. It shows that there is an inverted *U*-shaped relationship between environmental regulation and enterprise green technological innovation. This conclusion will not change due to the types of green technological innovation, while the impact of environmental regulation on enterprise green product innovation is greater than that of green process innovation. In addition, external financing constraints will reduce the impact of environmental regulation on enterprise green technological innovation. The research conclusions have certain reference value for deepening the understanding of green technological innovation and optimizing the relationship between government and market.

## 1. Introduction

Environmental pollution, resource security, and climate crisis have made countries around the world re-think the economic and social development paths [1,2,3]. The disadvantages of the traditional development model driven by resources and energy are gradually appearing, and more and more people realize that it is unsustainable. Examples of this are the London smog incident, Minamata in Japan, ozone layer destruction, global temperature rise, etc. With the deterioration of environmental problems, the current economic development model urgently needs a green transformation [4]. The green transformation may include four aspects: first, improve the efficiency of resources and energy to reduce the discharge of pollutants as much as possible [5]; second, establish an environment-friendly society to ensure biological diversity and maintenance [6]; third, perfect policy support [7]; fourth, actively develop a low-carbon economy to reduce greenhouse gas emissions to the atmospheric environment [8]. The development of green low-carbon circular economy cannot be driven by factors, and technological innovation will become the core driving force [7,9,10].

Green technological innovation follows the ecological principle, which can be defined from broad and narrow dimensions [11]. From the broad perspective, all technological innovations that can achieve resource conservation and environmental protection can be identified as green technological innovation [12,13]. From the narrow perspective, green technological innovation only represents the innovation in environmental technology, environmental processes, and environmental products in the whole process of product production or service provision [14,15]. Considering comprehensively, the narrow perspective of green technological innovation is closer to the research goal, which can be divided into green process innovation and green product innovation [16,17,18].

As the provider of market products or services, enterprises can effectively reduce the impact of their activities on the ecological environment by widely applying green technology [19,20]. However, due to the multiple externalities [21], technical uncertainty [22], market uncertainty, and imperfect management system of green technological innovation [23], enterprises lack the motivation and ability of innovation. In such a scenario, the adoption of some form of environmental regulation becomes a common choice for governments [24,25,26]. Environmental regulation includes administrative measures, legal measures, and economic measures, which can minimize the negative impact of enterprise production on the environment, resources and society, and achieved economic benefits [24]. Enterprise green technological innovation has the characteristics of high investment and high uncertainty. External financing is an important channel for enterprises to obtain green technological innovation funds [27,28]. Therefore, under the constraints of environmental regulation, part of the capital of enterprises will be occupied, and they will increasingly rely on external financing to invest in green technological innovation.

Based on the above description, we can find that environmental regulation may promote enterprise green technological innovation, but too strong environmental regulation may crowd out the capital for green technological innovation. This reason makes it necessary for us to focus on a certain type of enterprises, explore the relationship between environmental regulation and enterprise green technological innovation. Is there an impact boundary of environmental regulation on enterprise green technological innovation? Will the relationship between environmental regulation and enterprise green technological innovation change due to types of technological innovation? Will external financing constraints moderate the relationship between environmental regulation and enterprise green technological innovation? To solve these problems is of great significance to further optimize the relationship between government and market, and then stimulating green technological innovation of enterprises.

The rest of the article is organized as follows: In Section 2, we review the literature on the relationship among environmental regulation, green technological innovation, and external financing constraints. In Section 3, we describe our theoretical analysis and research hypothesis in detail. In Section 4, we give out our research design, including data source, model design, and variables measurement. Section 5 presents and discusses the empirical results. Section 6 presents the discussions and contributions. Section 7 concludes the paper.

## 2. Literature Review

### 2.1. Characteristics of Green Technological Innovation

Green technology can save resources, avoid or reduce environmental pollution, realize the recycling of raw materials and wastes, and thus solve the problems of excessive resource consumption and serious environmental pollution [29]. Green technological innovation can generally reduce cost of pollution control and operating, which will improve the competitiveness of enterprises [30]. For enterprises, green technological innovation, like other innovation activities, can bring long-term and sustained profit growth, and compared with other innovation activities, it can gradually improve the competitiveness of enterprises under the situation of stricter environmental regulation [20,31] However, there is a high substitution between green technological innovation and non-green technological innovation in product production. Choosing green technological innovation may result in giving up the initial productivity advantage of non-green technological innovation, which leads enterprises to face greater competitive pressure for a long period time.

It is precisely because of the positive externality that governments are actively promoting green technological innovation [32]. Firstly, the imitation pressure and knowledge spillover effect brought by green technological innovation can promote other enterprises in the same industry to participate in innovation activities [33] Secondly, green technological innovation can affect upstream and downstream industries through production standards, supply standards, and other ways, and then play a good radiation role [20]. Thirdly, green technological innovation can significantly improve the end-treatment capacity, and provide support for the pollution control and natural resource protection [34]. Fourthly, green technological innovation can also help improve the construction and maintenance of environmental laws and regulations, which will help to meet the requirements of environmental protection and green growth.

However, green technological innovation may have negative externalities of the inhibition effect on economy. In the short term, the regions where green technological innovation is carried out may face the investment and risks in the innovation process. It takes a longer time to fully adapt to the new development model [34]. In addition, green technological innovation may also have a crowding-out effect on other technological innovations and prevent non-green technological innovation paths [35]. Consequently, enterprises may be reluctant to carry out green technological innovation due to cost considerations and the negative effects [36].

### 2.2. Environmental Regulation and Green Technological Innovation

There are four views on the relationship between environmental regulation and green technological innovation: first, according to Porter’s hypothesis, some studies point out that stronger environmental regulation is beneficial to green technological innovation. It is considered that the cost of environmental regulation can be offset by the compensation effect of innovation [37,38]. Second, some scholars believe that environmental regulation increases the cost of enterprises from the perspective of neoclassical economics, so it will always inhibit the green technological innovation of enterprises [39,40]. Third, some studies argue that there is a nonlinear relationship between environmental regulation and technological progress due to the change of time scale [38]. In the short term, investment related to pollution control will crowd out investment in green technology research and development, but in the long term, the compensation effect of innovation can generate additional profits to promote technological progress [41]. Fourthly, some studies show that there is no obvious relationship between them. At present, the research findings are generally developed from the above viewpoints, which are often used to explain the relationship between the two in some specific situations. For example, Steinhorst and Matthies analyzed the energy industry, and found that environmental regulation can have a positive impact on green technological innovation by promoting public interest and investment [42]. Stucki et al proved that environmental regulation would crowd out the investment in green product innovation and adversely affect it [43]. Yuan et al reviewed the data of 28 manufacturing industries in China from 2003 to 2013 and found that environmental regulation has neither enhanced the ability of technological innovation nor improved the ecological efficiency [3].

The relationship between environmental regulation and green technological innovation may vary depending on the type of green technological innovation. Currently, an increasing number of studies are re-testing the relationship between environmental regulation and green technological innovation by subdividing the type of environmental regulation. It is the current research focus to further divide green technological innovation into green process innovation and green product innovation. For example, Song et al explored the impact mechanism of environmental regulation and other measures on green product innovation [44]. It is found that the impact of environmental regulation on green product innovation is closely related to its intensity, and there may be a *U*-shaped relationship. Liu et al. found that environment regulation can promote green process innovation, but the realization of this improvement depends on the specific level of government subsidies [45]. It means that under a certain intensity of subsidies, environment regulation can have a compensation effect on green process innovation. Some empirical results further show that there is a significant correlation between green process innovation and green product innovation [46,47]. In a word, the relationship between environmental regulation and green technological innovation is not unique.

### 2.3. External Financing Constraints and Green Technological Innovation

Financing constraints is one of the common reasons that restrict the development scale and speed [48,49]. External financing constraints mean that enterprises will rely more on internal cash flow, and the scale of available funds is relatively limited [50]. Compared with non-green technological innovation, green technological innovation, often requires a larger amount of investment and a longer return period of investment [51]. It makes it possible to face serious external financing constraints [48]. At present, there is abundant research on green technological innovation, but few of them explore its development conditions from the perspective of internal capital flow. According to the literature, the phenomenon of external financing constraints will restrict the green technological innovation of enterprises by blocking cash flow, meanwhile the constraint will gradually easing as environmental regulation continue to improve [50]. In addition, the study also found that, the ownership and scale of enterprises may affect the impact of external financing on green technological innovation. When the scale of enterprises are more than the threshold value, the significance of external financing on green technological innovation becomes more significant [52].

In short, the phenomenon of external financing constraints will limit the technological innovation of enterprise, especially green technological innovation [50,53]. However, enterprise green technological innovation can alleviate the external financing constraints by improving the market competitiveness, financial performance, and market value, as well as by cooperating with environmental regulation [51,54,55]. The moderation effect will be affected by the nature of the enterprise. External financing constraints are involved in financing green technology innovation, so in order to ensure the rationality of environmental regulation, the relationship between external financing constraint and green technological innovation should also be considered. It needs further exploration on how external financing constraints moderate the relationship between environmental regulation and enterprise green technological innovation.

It is obvious to see that the existing literature has paid great attention to the relationship among environmental regulation and enterprise green technological innovation and conducted qualitative and quantitative research based on different perspectives, methods, and data. However, there is no consensus on the relationship between environmental regulation and green technological innovation in the existing studies, with conclusions such as positive correlation, negative correlation, non-linear relationship, and non-correlation. Additionally, there is a lack of empirical tests that can be conducted on specific type of enterprises, especially private enterprises because of the large number and the large impact. Just as Meng et al. found that more than half of China’s CO_2_ emissions are from micro, small, and medium-sized enterprises. In related studies, external financing constraints are often regarded as the direct factors that affect enterprise green technological innovation [56]. There is still a lack of analysis on how external financing constraints moderate the relationship between environmental regulation and green technological innovation, which named indirect impact.

Therefore, our study will use the data of 2278 manufacturing enterprises to empirically test the inverted *U*-shaped relationship between environmental regulation and enterprise green technological innovation and compare the impact of environmental regulation on green process innovation and green product innovation, respectively. On this basis, we further analyze whether external financing constraints moderate the impact of environmental regulation on enterprise green technological innovation. The conclusions will provide reference for promoting green technological innovation by optimizing the relationship between government and market, and also provide policy implications for supporting high-quality development.

## 3. Research Hypothesis

### 3.1. Inverted U-Shaped Relationship between Environmental Regulation and Green Technological Innovation

According to institutional theory and innovation theory, the impact of government environmental regulation on enterprise green technological innovation has complied both innovation compensation effect and cost effect. The former is mainly reflected in the improvement of production technology and product quality, while the latter is mainly reflected in the capital crowding out effect. Porter hypothesis proposed that a certain environmental regulation although increasing firm’s production costs, but also force technological innovation of enterprise, thus resulting in the compensation effect [41,57,58]. The key to the effect of innovation compensation is whether the benefits of enterprise green technological innovation can make up for the investment [58]. An enterprise carries out green process innovation or green product innovation will not only effectively reduce the production cost, but also improve the product quality [59] and the market competitiveness [46]. Environmental regulation within a certain range of intensity has released signals to enterprises that green technological innovation is profitable [5]. Enterprises with high pollution emission and high energy consumption try to reduce emission intensity and energy use intensity by carrying out green technological innovation, so as to produce the effect of innovation compensation [60]. However, when the government environmental regulation surplus a threshold intensity, part of the capital of enterprises are forced to meet the sewage charges, crowding out the investment in updating production equipment, processes, and developing new products [40]. Generally speaking, enterprise green technological innovation belongs to the category of technological innovation, which can be divided into green process innovation and green product innovation [46,47]. Whether it is green process innovation or green product innovation, enterprise have to weigh the costs and benefits of innovation in order to maximize the benefits. Therefore, environmental regulation can have the direct impact on enterprise green technological innovation, the only difference is the impact magnitude.

Based on the above analysis, we put forward the following two assumptions:

**H_1_**. *There is an inverted U-shaped relationship between environmental regulation and enterprise green technological innovation*.

**H_2_**. *The inverted U-shaped relationship between environmental regulation and enterprise green technological innovation will not change due to the type of green technological innovation*.

### 3.2. External Financing Constraints Moderate the Relationship between Environment Regulation and Enterprise Green Technological Innovation

Green technological innovation, as a long-term and high-risk activity, needs sustained investment [61]. The practice in developed countries shows that equity financing and debt financing are important external financing ways to effectively make up for the shortage of funds for enterprise technological innovation [52]. Pecking order theory suggests that equity financing has the characteristic of chasing premium, which has a significant impact on the technological innovation behavior [62]. Debt heterogeneity theory implies that related creditors have the characteristics of innovation and tolerance and can achieve mutual win-win by promoting technological innovation [52]. The impact of external financing on enterprise green technological innovation largely depends on whether its value can meet the expected benefits of the investor. The main goal of equity financing is to obtain risk premium, and the main goal of debt financing is to obtain fixed income [52].

As mentioned above, green technological innovation has the characteristics of multiple externalities, technical uncertainty, and market uncertainty, which leads to the uncertainty of innovation input-output [63]. Meanwhile, due to the imperfection of property rights definition and market transaction mechanism, the environmental benefits and social benefits generated by green technological innovation cannot be transferred into economic benefits [64,65]. It also fails to meet the pursuit of risk premium in equity financing and the pursuit of stable income claim by creditors. With the tightening of external financing constraints, the capital availability of enterprises is seriously affected [50,66]. Without sufficient external funds, the investment in green technological innovation will decreases. Therefore, the impact of environmental regulation on green technological innovation will decline with the strengthening of external financing constraints. More specifically, compared with the situation with high external financing constraints, the environmental regulation with low external financing constraints has a more significant impact on green technological innovation.

Based on the above analysis, we put forward the following three assumptions:

**H_3_**. *External financing constraints play a negative moderating effect on the relationship between environmental regulation and enterprise green technological innovation*.

**H_4_**. *The moderating effect of external financing constraints on the relationship between environmental regulation and enterprise green technological innovation will not change with the type of green technological innovation*.

## 4. Research Design

### 4.1. Data Sources

**Figure 1:**
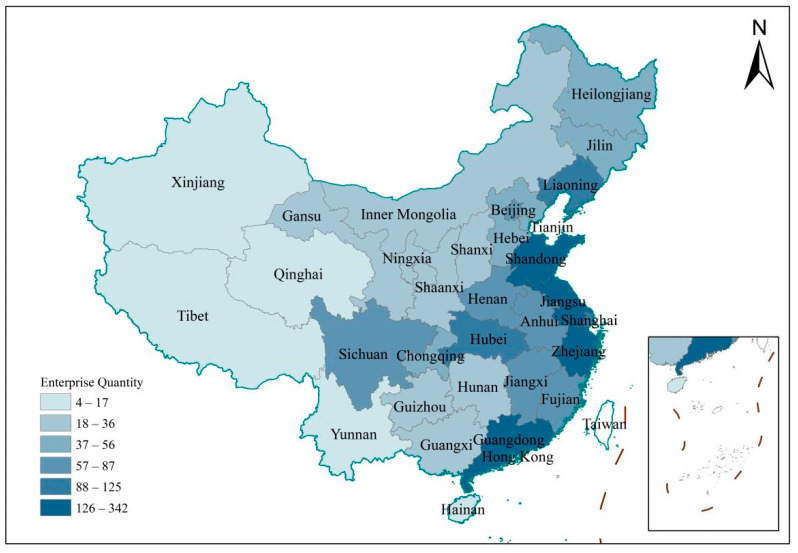
Spatial distribution of data.

The data used in this study comes from the 11th sample survey database of private enterprises in China. The survey was jointly conducted by the United Front Work Department of the CPC Central Committee, the All-China Federation of Industry and Commerce, the State Administration for Industry and Commerce and the China Private Economic Research Association. The survey was started in March 2014, and the survey method was obtained according to the random sampling proportion of 0.005%. Enterprises of different industries and sizes from 31 provinces participated in the questionnaire survey. The questionnaire consists of three parts: the situation of major investors, the situation of enterprises, and the development environment of enterprises, with a total of 36 types of questions. In total, 6500 questionnaires were actually distributed, and 6144 valid questionnaires were collected, with an effective recovery rate of 94.5%. Considering the characteristics of sample data distribution and enterprise green technological innovation, 2278 private enterprises with manufacturing industries in the primary, secondary, and tertiary industries are selected (In this survey, enterprises choose from three major industries; the first major industry is 1986 manufacturing industries, the second major industry is 210 manufacturing industries, and the third major industry is 82 manufacturing industries, with a total of 2278 enterprises, which is the industry with the largest number of enterprises.). The spatial distribution of enterprises is shown in Figure 1. It can be found that the eastern region and some central provinces are the main sources of sample enterprises. This result is also perfectly in line with the actual situation of China.

### 4.2. Variable Description

(1) Explained variables: consistent with previous studies, we set three explained variables: enterprise green technological innovation, enterprise green process innovation, and enterprise green product innovation. Enterprise green technological innovation is measured by “the total investment of enterprises for technological innovation, technological transformation, and new product R&D in 2013 (10,000 yuan)”, which is expressed as *GTI_T*. Enterprise green process innovation is measured by “the investment amount of enterprises for technological innovation and technological transformation in 2013 (10,000 yuan)”, which is expressed as *GTI_P*. Enterprise green product innovation is measured by “the investment amount of enterprises for new product R&D in 2013 (10,000 yuan)”, which is expressed as *GTI_Q*. In order to minimize the impact of outliers on model estimation, in the process of data processing, both ends of explanatory variables are end-processed by 1%, and the above three variables are included in the empirical test model in the form of logarithm.

(2) Explanatory variables: drawing from the previous research, the environmental regulation is measured by “environmental protection and pollution control fees paid by enterprises in 2013 (10,000 yuan)”, which is expressed as *ERI*. Considering that there may be a “*U*-shaped” or “inverted *U-*shaped” relationship between environmental regulation and enterprise green technological innovation, the square term of environmental regulation is also included as an explanatory variable in this study, which is expressed as *ERI_2*. In the same way as the explained variables, the environmental protection and pollution control fees paid by enterprises are end-processed by 1% at both ends, and then included in logarithmic form.

(3) Moderator variables: external financing, as an important channel for enterprises to obtain funds, has affected the investment of enterprises in green technological innovation. Therefore, the external financing constraints of enterprises will have a moderating effect on the relationship between environmental regulation and green technological innovation. According to the literature, the interaction term between enterprise asset liability ratio (expressed as *EFR*) at the end of 2013 and environmental regulation is used to judge whether there is a moderating effect.

(4) Control variables: enterprise organization, enterprise managers, and external development environment are select as three control dimensions to improve the accuracy of the research hypothesis test. First of all, the variables of organizational dimension are included enterprise age (*E_AGE)*, number of employees (*LAB*), sales profit rate (*ROS*) and annual employee training fee (*TRAIN*). Secondly, the variables of enterprise mangers’ dimension mainly are controlled by entrepreneur’s age (*EAG*), entrepreneur’s gender (*GEND*), entrepreneur’s education level (*EDU*), entrepreneur’s political outlook (*PLO*), and entrepreneur’s political identity (*PLS*). Finally, the variables of external development environment dimension of enterprises are controlled by enterprise development environment (*ENE*) and enterprises’ overseas investment (*EXT*). The definitions and measurement methods of each variable are shown in Table 1.

### 4.3. Model Design

This study tests the impact of environmental regulation on green technological innovation. Specifically, it includes three types of explanatory variables: green technological innovation (Model 1), green process innovation (Model 2), and green product innovation (Model 3) to verify whether the impact changes with the type of green technological innovation. In addition, in Model 4 to Model 6, the moderating effect of external financing constraints of enterprise is studied by adding interaction term. As the explained variable, explanatory variable and moderate variable are all continuous variables, the following model can be established:

(Model 1) $$\begin{array}{cc} GTI\_T= & \beta_{0}+\beta_{1}ERI+\beta_{2}ERI\_2+\beta_{3}E\_AGE+\beta_{4}LAB+\beta_{5}AGE+\beta_{6}GEND+\\ \; & \beta_{7}EDU+\beta_{8}ROS+\beta_{9}ENE+\beta_{10}PLO+\beta_{11}EXT+\beta_{12}TRAIN+\beta_{13}PLS+\varepsilon \end{array}$$

(Model 2) $$\begin{array}{cc} GTI\_P= & \beta_{0}+\beta_{1}ERI+\beta_{2}ERI\_2+\beta_{3}E\_AGE+\beta_{4}LAB+\beta_{5}AGE+\beta_{6}GEND+\\ \; & \beta_{7}EDU+\beta_{8}ROS+\beta_{9}ENE+\beta_{10}PLO+\beta_{11}EXT+\beta_{12}TRAIN+\beta_{13}PLS+\varepsilon \end{array}$$

(Model 3) $$\begin{array}{cc} GTI\_Q= & \beta_{0}+\beta_{1}ERI+\beta_{2}ERI\_2+\beta_{3}E\_AGE+\beta_{4}LAB+\beta_{5}AGE+\beta_{6}GEND+\\ \; & \beta_{7}EDU+\beta_{8}ROS+\beta_{9}ENE+\beta_{10}PLO+\beta_{11}EXT+\beta_{12}TRAIN+\beta_{13}PLS+\varepsilon \end{array}$$

(Model 4) $$\begin{array}{cc} GTI\_T= & \gamma_{0}+\gamma_{1}ERI+\gamma_{2}ERI\_2+\gamma_{3}EFR+\gamma_{4}EFR\times ERI+\gamma_{5}EFR\times ERI\_2+\\ \; & \gamma_{6}E\_AGE+\gamma_{7}LAB+\gamma_{8}AGE+\gamma_{9}GEND+\gamma_{10}EDU+\gamma_{11}ROS+\\ \; & \gamma_{12}ENE+\gamma_{13}PLO+\gamma_{14}EXT+\gamma_{15}TRAIN+\gamma_{16}PLS+\varepsilon \end{array}$$

(Model 5) $$\begin{array}{cc} GTI\_P= & \gamma_{0}+\gamma_{1}ERI+\gamma_{2}ERI\_2+\gamma_{3}EFR+\gamma_{4}EFR\times ERI+\gamma_{5}EFR\times ERI\_2+\\ \; & \gamma_{6}E\_AGE+\gamma_{7}LAB+\gamma_{8}AGE+\gamma_{9}GEND+\gamma_{10}EDU+\gamma_{11}ROS+\\ \; & \gamma_{12}ENE+\gamma_{13}PLO+\gamma_{14}EXT+\gamma_{15}TRAIN+\gamma_{16}PLS+\varepsilon \end{array}$$

(Model 6) $$\begin{array}{cc} GTI\_Q= & \gamma_{0}+\gamma_{1}ERI+\gamma_{2}ERI\_2+\gamma_{3}EFR+\gamma_{4}EFR\times ERI+\gamma_{5}EFR\times ERI\_2+\\ \; & \gamma_{6}E\_AGE+\gamma_{7}LAB+\gamma_{8}AGE+\gamma_{9}GEND+\gamma_{10}EDU+\gamma_{11}ROS+\\ \; & \gamma_{12}ENE+\gamma_{13}PLO+\gamma_{14}EXT+\gamma_{15}TRAIN+\gamma_{16}PLS+\varepsilon \end{array}$$

where $\beta_{0}$ and $\gamma_{0}$ represent intercept term; $\beta_{1}$ and $\gamma_{1}$ are the regression coefficients of the first term of environmental regulation; $\beta_{2}$ and $\gamma_{2}$ are the regression coefficients of the quadratic term of environmental regulation; $\gamma_{3}$ is the regression coefficient of external financing constraints; $\gamma_{4}$ is the regression coefficient of the intersection term of external financing constraints and environmental regulation; $\gamma_{5}$ represents the regression coefficient of the intersection of the external financing constraints and the quadratic term of environmental regulation; $\beta_{3}∼\beta_{13}$ and $\gamma_{6}∼\gamma_{16}$ are the regression coefficients of the control variables, respectively; ε is independent and identically distributed random disturbance term.

## 5. Empirical Tests

### 5.1. Descriptive Statistic

The definitions, measurements, and descriptive statistics of dependent variables, independent variables, moderator variable, and control variables are shown in Table 1. It can be seen that the mean of green process innovation is −2.730, with the median value of −9.210 and the standard deviation is 6.899. The mean value of green product innovation is −3.081, with a median and a standard deviation of −9.210 and 6.862, respectively. It can be seen that despite the logarithmic process of these two variables, there is a big gap between the median and standard deviation, as well as the maximum and minimum values, which indicates that some enterprises still invest less in green process innovation and green product innovation. The mean value of green technological innovation is −1.774, with a median and a standard deviation of 2.079 and 7.159, respectively. The gap between the maximum and minimum of environmental regulation is relatively small, indicating that the situation that enterprises paying sewage charges is common. In terms of external financing constraints, there is a large gap between the minimum value and the maximum value, ranging from 0 to 500%, indicating that there is a large difference in the enterprise’s asset liability ratio.

In terms of control variables, the longest age of enterprise is 41 years, with an average of 10 years. The average age of entrepreneurs is 47, of which the oldest is 68 and the youngest is 16. The gender of entrepreneurs reflects that most of them are men, accounting for 88.8%. The education level of entrepreneurs shows that most entrepreneurs are college graduates or above.

The proportion of the entrepreneurs who are members of the CPC is 41.4%, and the proportion of deputies to the National People’s Congress at all levels or CPPCC is 38.2%. The standard deviation of enterprise development environment exceeds 2, indicating that the external environment is still strict, which will have a certain impact on enterprise green technological innovation.

Based on descriptive statistics, this study further analyzes the Pearson correlation of each variable, as shown in Table 2. The results show that there is a significant positive correlation between environmental regulation and enterprise green technology innovation, which also provides directional support for the subsequent hypothesis test. Therefore, we speculate that the external financing constraints may affect the investment of green technological innovation as a moderator variable.

### 5.2. Regression Results

Before hypothesis testing, this study tested the multicollinearity of six models. The results show that the Variance Inflation Factor (*VIF*) of all models does not exceed 2, indicating that there is no serious multicollinearity problem in the models. From the descriptive statistical results in Table 2, it can be found that there were no variables with particularly high correlation. Table 3 shows the results of the impact of environmental regulation on enterprise green technological innovation. The dependent variable of Model 1 is the logarithm of the sum of the total investment of enterprises for technological innovation, technological transformation, and new product R&D. The dependent variable of Model 2 is the logarithm of the investment of enterprises in technological innovation and technological transformation. The dependent variable of Model 3 is enterprise green product innovation. Except for the dependent variable, the control variables of the three models are the same.

Model 1 shows that the environmental regulation coefficient is 0.516 and the square term coefficient of environmental regulation is −0.463, both of which are significant at the statistical level of 1%. With the gradual tightening of environmental regulation, the investment in enterprise green technological innovation will first increase, and when the environmental regulation exceeds a certain extent, the investment in enterprise green technological innovation will decrease. This shows that the impact of environmental regulation on enterprise green technological innovation is inverted *U*-shaped. In other words, there is an optimal regulation intensity for promoting green technological innovation. The above conclusions are still valid for green process innovation and green product innovation. For enterprise green process innovation (Model 2 and Model 3), the environmental regulation coefficient and its square term are 0.372 and −0.285, respectively, and both are significant at the statistical level of 10%. For enterprise green product innovation, the environmental regulation coefficient and the square of environmental regulation are 0.479 and −0.428, respectively, both of which are significant at the statistical level of 1%. The above results show that the impact of environmental regulation on enterprise green technological innovation is inverted *U*-shaped, and there is no difference when the types of green technological innovation change. Therefore, the research hypothesis that H_1_ and H_2_ are supported by research data.

### 5.3. Analysis of Moderating Effect

Based on the benchmark regression results, it can be judged that there is a nonlinear relationship between government environmental regulation and enterprise green technological innovation. At the same time, many existing studies have also found that the external financing constraints of enterprises will also have an impact on their green technological innovation. However, whether it has an indirect effect is not studied yet. To this end, we further investigate the moderating effect of external financing constraints on the relationship between environmental regulation and enterprise green process innovation. Model 4, Model 5, and Model 6 are built by adding the external financing constraints of enterprises, the interaction term of external financing constraints and environmental regulation, and the interaction term of square term of environmental regulation and external financing constraints. The empirical results are reported in Table 4.

The empirical results show that: (1) The external financing constraints of enterprises have a negative impact on green technological innovation, green process innovation, and green product innovation. Results show that the stricter the external financing is, the lower the willingness of enterprises to invest in green technological innovation. (2) The interaction term coefficients of external financing constraints and environmental regulation in Model 4, Model 5, and Model 6 are significant statistical level of 1%. The results show that external financing constraints will offset the promotion effect of environmental regulation on green technological innovation. (3) The interaction term coefficients of external financing constraints and the square of environmental regulation is significant at the statistical level of 1%. The results show that external financing constraints will slow down the growth range of promotion of environmental regulation on green technological innovation, that is, with the strengthening of external financing constraints, the impact of environmental regulation on green technological innovation becomes more and more gentle.

### 5.4. Robustness Test

In the above research, we took manufacturing enterprises as the research object, empirically tested the impact of environmental regulation on enterprise green technological innovation. In order to enhance the robustness of the research, the moderating effect analysis is repeated in all industries enterprises. The empirical results are shown in Table 5. As can be seen, there is no significant change. Compared with the benchmark results, external financing constraints have no significant effect on green technological innovation, green process innovation and green product innovation; that is, external financing constraints will not directly affect the enterprise green technological innovation, but to promote green technological innovation by indirectly affecting the environmental regulation.

## 6. Discussion

This study has the following theoretical contributions: firstly, previous studies have focused on the relationship between environmental regulation and green technological innovation at macro-level or industry-level [37,67] As the main agent facing the market, enterprises can not only meet the production environment standards, as well as the diversified needs of the market through green process improvement and new product development. Taking private enterprises in manufacturing industry as samples, the impact of environmental regulation on enterprise green technological innovation is studied, which is the expansion and enrichment of macro and industry scale research.

Secondly, as for the relationship between environmental regulation and green technological innovation, most of the existing studies focus on answer whether environmental regulation have an impact on enterprise green technological innovation [68], but there is no systematic answer to the impact extent, direction and heterogeneity. The nonlinear relationship between the environmental regulation and green technological innovation is verified; that is, environmental regulation has a positive impact on green technological innovation, and the extent of the impact is decreasing. The possible reason for this result is that there is an optimal value in the role of government environmental regulation. By releasing signals to encourage enterprises to adopt innovative behaviors, they can create more economic value while meeting the legitimacy of production.

Thirdly, most research focuses on the direct effect of external financing constraints on enterprise green technological innovation, but the analysis of indirect effects is not discussed thoroughly [50]. On the basis of demonstrating the relationship between environmental regulation and green technological innovation, the moderating effect of external financing constraints on green technological innovation is tested. The results confirm that external financing constraints will decrease the promotion of environmental regulation on green technological innovation, and with the stricter external financing constraints, the impact will be reduced to a certain extent. The result is largely due to the fact that external financing constraints affect the channels for enterprises to obtain funds to a certain extent and then affect the impact of environmental regulation on green technological innovation

Finally, many studies emphasized the need to build a market-oriented green technological innovation system [21], use market-oriented regulations to encourage enterprises to adopt green technological innovation behaviors, or ensure the performance of green technological innovation. However, this conclusion lacks empirical data testing. We comprehensively consider the impact of the combination of government environmental regulation and external financing constraints on enterprise green technological innovation and provide a new perspective for analyzing and optimizing the relationship between government and market. This means that in the future, in the process of promoting green technological innovation, we should make full use of the role of government regulation and the supporting role of market mechanism to realize the combination of a government and an effective market.

Through the above research, we can obtain the following implications: first, we should continue to strengthen environmental regulation, despite the current downward pressure on the economy. Due to the impact of the COVID-19 epidemic, the economic growth rate has further slowed down. In this background, if we try to stimulate the economy by means of reducing the environmental regulation or allowing high-carbon projects, this will have a negative impact on green economic recovery in the long run. According to Porter’s hypothesis, appropriate enterprise environmental regulation will stimulate enterprises’ green technological innovation behavior, thus offsetting the environmental cost of production and ultimately improving the market competitiveness of enterprises. The research conclusion of this study further confirms this theoretical hypothesis. Therefore, it is not the appropriate time to reduce the environmental regulation, but to focus the design and implementation of regulatory measures on driving the enterprise green technological innovation.

Secondly, external financing constraints should be reduced as much as possible to meet the capital needs of enterprise green technological innovation. Only relying on government funds to promote enterprise green technological innovation is not an efficient and sustainable way. Effectively reducing external financing constraints and making good use of social capital are the key to high-quality technology innovation. Therefore, we should speed up the construction of technology and finance systems to promote enterprise green technological innovation and set up an information management platform for enterprise green technological innovation. We should, additionally, give attention to the role of venture capital, institutional investors, and other financial entities, promote the effective combination of green technology and finance, and effectively alleviate the external financing constraints of enterprise green technological innovation.

Finally, we should make good use of both government and market tools to promote green technological innovation of enterprises. In promoting green technological innovation, there is no perfect market mechanism. The government should take appropriate measures to intervene in market failure caused by dual externalities. However, excessive intervention will lead to government failure. To keep a balance, it is necessary to organically integrate the functions of government and market. On the one hand, it is necessary for the government to exert pressure on production enterprises by implementing environmental regulation, and on the other hand, it is necessary to continuously optimize the market environment to improve the allocation efficiency of innovation elements. The conclusion also confirms that increasing the environmental regulation and reducing external financing constraints can further stimulate the investment of enterprises in green technological innovation.

There are some limitations in this research, which need to be further improved in the future. First, regarding variable measurement, this study selects the investment amount of technological innovation, technological transformation, and new product R&D of enterprises to represent green process innovation and green product innovation, respectively, and further focus on innovation investment related to environmental improvement is needed in the future. Second, our study uses cross-sectional data. Future research should test the results by the panel data of listed companies. Thirdly, the specific transmission path needs to be further explored, for example, the increase of production cost, the improvement of market entry threshold and green reputation mechanism, etc.

## 7. Conclusions

Based on 2278 manufacturing enterprises data of private enterprises in China, the nonlinear relationship between environmental regulation and green technological innovation, green process innovation, and green product innovation are investigated. On this basis, the moderating effect of external financing constraints on the nonlinear relationship between environmental regulation and green technological innovation is investigated and the robustness is tested by the data of enterprises in the whole industry. The following conclusions can be drawn: (1) Government environmental regulation can positively stimulate enterprise green technological innovation and will not change due to the type of green technological innovation. (2) There is an inverted *U*-shaped relationship between the environmental regulation and enterprise green technological innovation; that is, with the improvement of the environmental regulation, the investment in enterprise green technological innovation keeps increasing, but the increasing range keeps decreasing for each unit increase in environmental regulation. (3) The stricter the external financing constraints is, the lower the willingness of enterprises to innovate in green technology. External financing constraints will reduce the promotion of environmental regulation on green technological innovation, and with the strengthening of external financing constraints, the promotion of environmental regulation on green technological innovation will be reduced to some extent.

## Figures and Tables

**Table 1:** Definition and statistical characteristics of variables.

Variable Name	Variable Definition	Sample Size	Mean	Standard Deviation	Minimum Value	Median Value	Maximum Value
Green process innovation	Logarithms of investment in technological innovation and technological transformation in 2013.	1984	−2.730	6.899	−9.210	−9.210	8.161
Green product innovation	Logarithms of new product R&D investment in 2013.	1942	−3.081	6.862	−9.210	−9.210	8.471
Green technological innovation	Logarithms of investment in technological innovation, technological transformation and new product R&D in 2013.	1877	−1.774	7.159	−9.210	2.079	8.923
Environmental regulation	Logarithms of environmental protection and pollution control fees paid in 2013.	1956	−3.836	5.378	−9.210	−1.609	5.799
External financing constraints	Asset-liability ratio of enterprises at the end of 2013 (%).	1220	44.922	30.835	0.000	43.000	500.000
Enterprise history (year)	2013 minus the enterprise establish year and then take the logarithm.	2194	2.308	0.928	−9.210	2.485	3.714
Number of employees in enterprise	In 2013, the number of employees employed by enterprises and then take the logarithm.	2241	4.677	1.610	−9.210	4.736	10.309
Entrepreneur’s age (year)	2013 Subtract the entrepreneur’s birth year and then take the logarithm.	2228	3.825	0.187	2.708	3.850	4.220
Entrepreneur’s gender	The gender is set to 1 for men and 0 for women.	2269	0.888	0.315	0.000	1.000	1.000
Entrepreneur’s education	1 for primary school and below, 2 for junior high school, and so on, 6 for graduate students.	2246	4.037	1.113	1.000	4.000	6.000
Enterprise sales profit rate	Ratio of sales profit to operating income of enterprises in 2013	2034	−0.021	3.912	−17.516	0.044	0.940
Enterprise development environment	The development environment of enterprises has improved in the past two years.	2257	3.264	1.057	1.000	4.000	5.000
Political features of entrepreneurs	Enterprise members of the Communist Party of China are 1, and the rest are 0.	2278	0.382	0.486	0.000	0.000	1.000
Foreign investment of enterprises	Logarithms of enterprises’ overseas investment in 2013.	2047	−8.803	2.367	−9.210	−9.210	8.343
Enterprise annual staff training fee	Logarithms of annual employee training expenses of enterprises in 2013.	2016	0.143	4.035	−6.908	1.610	8.517
Entrepreneur’s political identity	Member of the National People’s Congress or CPPCC are 1, and the rest are 0.	2278	0.414	0.493	0.000	0.000	1.000

**Table 2:** Correlation coefficient of main variables.

	1	2	3	4	5	6	7	8	9	10	11	12	13	14	15	16
*GTI_P*	1.00															
*GTI_Q*	-	1.00														
*GTI_T*	-	-	1.00													
*ERI*	0.18 ***	0.16 ***	0.16 ***	1.00												
*EFR*	−0.00	0.00	0.02	−0.03	1.00											
*E_AGE*	0.05	0.04	0.05	0.09 ***	0.02	1.00										
*LAB*	0.32 ***	0.29 ***	0.34 ***	0.33 ***	0.04	0.15 ***	1.00									
*AGE*	0.05	0.01	0.06	0.00	0.11 ***	0.28 ***	0.09 ***	1.00								
*GEND*	−0.00	−0.03	−0.03	−0.02	−0.03	−0.04	−0.09 ***	−0.07 **	1.00							
*EDU*	0.11 ***	0.16 ***	0.15 ***	0.06 *	−0.04	−0.02	0.23 ***	−0.16 ***	−0.03	1.00						
*ROS*	0.12 ***	0.09 ***	0.11 ***	−0.03	−0.10 ***	−0.00	0.04	−0.04	0.01	−0.06	1.00					
*ENE*	0.12 ***	0.10 ***	0.10 ***	0.12 ***	−0.11 ***	−0.04	0.14 ***	−0.07 **	−0.03	0.13 ***	0.08 **	1.00				
*PLO*	0.12 ***	0.10 ***	0.13 ***	0.16 ***	−0.02	0.09 ***	0.16 ***	0.17 ***	−0.10 ***	0.14 ***	−0.01	0.07 **	1.00			
*EXT*	0.12 ***	0.10 ***	0.11 ***	0.09 ***	0.02	0.09 **	0.13 ***	0.03	0.01	0.00	0.04	−0.00	0.06	1.00		
*TRAIN*	0.39 ***	0.40 ***	0.43 ***	0.27 ***	−0.02	0.11 ***	0.46 ***	0.00	−0.03	0.22 ***	0.06 *	0.18 ***	0.16 ***	0.11 ***	1.00	
*PLS*	0.16 ***	0.13 ***	0.15 ***	0.21 ***	0.00	0.15 ***	0.25 ***	0.12 ***	−0.06 *	0.12 ***	−0.04	0.05	0.19 ***	0.07 **	0.20 ***	1.00

Note: * *p* < 0.10, ** *p* < 0. 05 and *** *p* < 0.01.

**Table 3:** Impact of environmental regulation on enterprise green technological innovation.

Variables	Green Technological Innovation	Green Process Innovation	Green Product Innovation
	Model 1	Model 2	Model 3
Environmental regulation	0.516 ***	0.372 *	0.479 **
	(0.199)	(0.195)	(0.197)
Square of environmental regulation	−0.463 ***	−0.285 *	−0.428 **
	(0.173)	(0.170)	(0.172)
Enterprise history (year)	−0.004	−0.033	0.039
	(0.137)	(0.133)	(0.126)
Number of employees in enterprise	0.822 ***	0.720 ***	0.739 ***
	(0.170)	(0.161)	(0.127)
Entrepreneur’s age (year)	0.514	0.759	0.154
	(1.019)	(0.970)	(0.982)
Entrepreneur’s gender	−0.382	−0.222	−0.227
	(0.542)	(0.520)	(0.514)
Entrepreneur’s education	0.199	0.012	0.420 ***
	(0.163)	(0.155)	(0.161)
Enterprise sales profit rate	0.013 **	0.008	0.006
	(0.005)	(0.006)	(0.005)
Enterprise development environment	0.160	0.144	0.180
	(0.159)	(0.154)	(0.155)
Political features of entrepreneurs	0.586	0.504	0.209
	(0.362)	(0.347)	(0.347)
Foreign investment of enterprises	0.226 **	0.211 **	0.170 *
	(0.090)	(0.087)	(0.090)
Enterprise annual staff training fee	0.575 ***	0.499 ***	0.536 ***
	(0.047)	(0.044)	(0.041)
Entrepreneur’s political identity	0.507	0.538	0.353
	(0.359)	(0.349)	(0.350)
constant term	−6.245	−7.245 *	−7.468 *
	(4.178)	(4.040)	(4.043)
*R* ^2^	0.223	0.196	0.207
Adjusted *R*^2^	0.216	0.189	0.200
*F*	41.454	34.890	40.685
*N*	1431	1487	1466

Standard errors in parentheses. * *p* < 0. 10, ** *p* < 0. 05, *** *p* < 0. 01.

**Table 4:** Moderating effect of external financing constraints on the relationship between environmental regulation and enterprise green process innovation.

Variables	Green Technological Innovation	Green Process Innovation	Green Product Innovation
	Model 4	Model 5	Model 6
Environmental regulation	1.897 ***	1.908 ***	1.974 ***
	(0.459)	(0.486)	(0.424)
Square of environmental regulation	−1.728 ***	−1.678 ***	−1.772 ***
	(0.391)	(0.413)	(0.362)
External financing constraints	−0.017 *	−0.025 *	−0.024 **
	(0.013)	(0.015)	(0.011)
External financing constraints × environmental regulation	−0.026 ***	−0.029 ***	−0.029 ***
	(0.009)	(0.010)	(0.008)
External financing constraints× square of environmental regulation	0.025 ***	0.027 ***	0.027 ***
	(0.008)	(0.008)	(0.007)
Enterprise history (year)	−0.295	−0.304	−0.155
	(0.234)	(0.238)	(0.242)
Number of employees in enterprise	0.818 ***	0.761 ***	0.589 ***
	(0.179)	(0.182)	(0.167)
Entrepreneur’s age (year)	1.866	1.911	1.013
	(1.285)	(1.253)	(1.285)
Entrepreneur’s gender	−0.036	0.538	−0.248
	(0.691)	(0.676)	(0.680)
Entrepreneur’s education	0.342 *	0.168	0.528 **
	(0.202)	(0.199)	(0.207)
Enterprise sales profit rate	4.322 ***	5.017 ***	3.885 ***
	(1.558)	(1.593)	(1.476)
Enterprise development environment	0.127	0.259	0.153
	(0.209)	(0.207)	(0.212)
Political features of entrepreneurs	0.444	0.361	0.222
	(0.458)	(0.451)	(0.455)
Foreign investment of enterprises	0.161 *	0.132	0.127
	(0.092)	(0.099)	(0.097)
Enterprise annual staff training fee	0.645 ***	0.541 ***	0.599 ***
	(0.066)	(0.064)	(0.060)
Entrepreneur’s political identity	0.417	0.575	0.244
	(0.451)	(0.450)	(0.456)
constant term	−10.678 **	−12.201 **	−8.536
	(5.193)	(5.145)	(5.271)
*R* ^2^	0.251	0.216	0.214
Adjusted *R*^2^	0.236	0.202	0.199
*F*	24.960	21.008	20.954
*N*	846	881	875

Standard errors in parentheses. * *p* < 0. 10, ** *p* < 0. 05, *** *p* < 0. 01.

**Table 5:** Robustness test results.

Variables	Green Technological Innovation	Green Process Innovation	Green Product Innovation
	Model 1	Model 2	Model 3
Environmental regulation	1.726 ***	1.571 ***	1.621 ***
	(0.389)	(0.380)	(0.341)
Square of environmental regulation	−1.424 ***	−1.264 ***	−1.338 ***
	(0.337)	(0.329)	(0.294)
External financing constraints	−0.013	−0.017	−0.012
	(0.012)	(0.012)	(0.009)
External financing constraints × environmental regulation	−0.020 **	−0.021 ***	−0.020 ***
	(0.008)	(0.008)	(0.007)
External financing constraints× square of environmental regulation	0.019 ***	0.020 ***	0.019 ***
	(0.007)	(0.007)	(0.006)
Control variable	Controlled	Controlled	Controlled
	-	-	-
constant term	−2.439	−4.116	−2.140
	(3.719)	(3.489)	(3.514)
*R* ^2^	0.204	0.196	0.174
Adjusted *R*^2^	0.196	0.188	0.166
*F*	38.269	36.323	31.061
*N*	1701	1767	1747

Standard errors in parentheses. ** *p* < 0. 05, *** *p* < 0. 01.

## Data Availability

The datasets generated and/or analyzed during this study are available from the corresponding authors on reasonable request.
